# For Indirect Orthodontic Attachment Placement, Adding a Custom Composite Resin Base Is Not Beneficial: A Split-Mouth Randomized Clinical Trial

**DOI:** 10.1155/2022/9059697

**Published:** 2022-06-15

**Authors:** Mohamed S. Hassan, Fatma A. Abdelsayed, Amany H. Abdelghany, Zac Morse, Mai H. Aboulfotouh

**Affiliations:** ^1^Department of Orthodontics and Dentofacial Orthopedics, Egyptian Russian University, Cairo, Egypt; ^2^Department of Orthodontics and Dentofacial Orthopedics, Cairo University, Cairo, Egypt; ^3^Department of Oral Health, Auckland University of Technology, Auckland, New Zealand

## Abstract

**Aim:**

The aim of this study was to compare the chairside time, bond failure rate, and accuracy of bonding between two orthodontic attachment indirect bonding techniques.

**Methods and Materials:**

Two indirect bonding techniques were studied: unaltered base attachment (UA) and custom base attachment (CBA) methods. Eighty-four orthodontic attachments were bonded on six patient stone models. Preoperative models were digitally scanned, and subsequently, attachments were transferred with the aid of a single but sectioned vacuum-formed tray to their corresponding patients. Finally, participants were scanned after attachment bonding to make the postoperative digital replicas. Chairside time and immediate bond failure rates were measured and compared between both techniques. Postoperative and preoperative digital models were then superimposed in order to measure the accuracy of bonding in the three dimensions of space.

**Results:**

No differences existed between the two techniques regarding chairside time (*P*=0.87) and bond failure rates (*P*=0.37). There were also no differences found for the total attachment movement (*P*=0.73), mesiodistal (*P*=0.10), occlusogingival (*P*=0.31), torquing (*P*=0.21), and rotational measurements (*P*=0.18). The UA technique, however, proved to be more accurate for buccopalatal linear directions (*P*=0.04), whilst the CBA technique showed more accuracy for tipping angular deviations (*P* < 0.01). There was a statistically significant directional bias for the UA towards the occlusal (*P* < 0.01) and palatal (*P*=0.02) directions with mesial-out angular deviation (*P*=0.02).

**Conclusion:**

The two indirect bonding techniques were comparable for chairside time, bond failure rates, and most linear and angular measurements. The UA technique was, however, superior in buccopalatal directions, while the CBA method showed more tipping accuracy. Both techniques were efficient and reliable for indirect bonding.

## 1. Introduction

An accurate orthodontic attachment position is a fundamental factor in maximizing the treatment outcome benefit. Non-optimal placement of orthodontic attachments may lead to undesirable tooth movements, such as deviations in rotations, intrusion, and extrusion, in and out, tipping, or torque [1]. The placement of orthodontic attachments is accomplished by either one of the two majorly recognized approaches, namely, the one-stage direct or two-stage indirect bonding method. The indirect method was first introduced by Silverman et al. [2] and gained popularity due to some significant advantages, such as greater visibility during attachment positioning, improved patient comfort, and decreased chair time [3–5]. Some restrictions, however, related to the accuracy of transfer and errors in clinical bonding could be considered challenging drawbacks.

Most studies compared the accuracy between direct and indirect bonding approaches [1,4,6,7]; there is limited literature, however, on studies reporting the precision of different indirect bonding techniques. For instance, Castilla et al. and Schmid et al., in their in vitro trials, found superior accuracy compared to a single sheet transfer tray when using combined silicone-vacuum-formed trays and silicone trays, respectively [8, 9]. Moreover, Grunheid et al. [10] also found higher precision when using polyvinyl siloxane trays with an in vivo concept, and this was further supported by Möhlhenrich et al. [11] where they claimed promising results with the double polyvinyl siloxane trays.

More recently, with the aid of intraoral scanning, 3D printing, and digital treatment planning, many studies have been developed utilizing digital methods for indirect bonding. For example, Chaudhary et al. [12] found superior results for the 3D printed transfer trays when compared to traditional ones in almost all dimensions of space. In addition, a clinical study by Bachour et al. [13] demonstrated high transfer accuracy for 3D trays for linear dimensions. Of note is that all the previous trials adopted custom composite resin base attachments, whilst different transfer trays were examined.

There was a gap in evidence if adding a customized composite resin base to orthodontic attachments during the laboratory stage has additional benefits during clinical indirect bonding, compared to using an unaltered base. Thus, the purpose of this randomized clinical trial was to measure and compare both techniques in terms of (1) the chairside time, (2) immediate bond failure rate, and (3) the accuracy of clinical bonding in the three dimensions of space.

The null hypothesis is that there is no difference in adding custom composite resin base to the orthodontic attachments in the indirect bonding technique with regard to chairside time, bond failure, and accuracy of bonding. The hypothesis tests were carried out at a *P* value set at 0.05.

## 2. Methods and Materials

The trial was performed at Cairo University and approved by the Faculty of Dentistry Research Ethics Committee. The study was registered with ClinicalTrials.gov with an identifier number: NCT03365232. Prior to the commencement of the study, all participants or their legal guardians were acquainted with the investigation and provided written consent. There were no changes in the study design after the trial commencement.

### 2.1. Sample Grouping and Preparation

The R statistical package, version 3.3.1, was used to calculate the sample size. Paired *t*-test power calculation was used to detect the appropriate sample size. Mean differences and standard deviations were estimated, according to the study by Grunheid et al., based on the accuracy of measurements [10]. A total sample size of 56 teeth (four participants) was adequate to detect a mean difference in attachment placement accuracy between study groups of 0.007 mm (SD = 0.009), with a power of 95% and a two-sided significance level of 5%, with equal allocation to two arms. The sample size was increased to 84 teeth, 42 per group (i.e., six participants), to take into consideration the potential sample attrition.

### 2.2. Trial Design

The current study was a split-mouth randomized controlled clinical trial to eliminate selection bias and interparticipant variability, with a 1 : 1 allocation following the CONSORT statement reporting guidelines [14].

### 2.3. Participants, Randomization, and Eligibility Criteria

Fifteen patients were assessed for eligibility according to the inclusion and exclusion criteria in the two-month recruitment period. Nine participants were excluded because they did not fit the eligibility criteria, and hence, six participants (84 teeth, 42 per group) were recruited from the Department of Orthodontics, Cairo University, Cairo, Egypt. The inclusion criteria for trial participants were as follows:

1. Patients with an age range from 15 to 25 years old requiring nonextraction fixed orthodontic therapy
2. Participants having a full set of sound maxillary permanent teeth
3. Space problems ranging from moderate spacing to mild crowding using the Crowding index (CI)
4. Good oral hygiene measures using plaque and gingival indices

Exclusion criteria were as follows:

1. Patients having signs of caries
2. Participants having extensive restorations involving more than two tooth surfaces or labial and buccal surface restorations
3. Patients having signs of fluorosis or enamel hypoplasia, previous orthodontic treatment, or those requiring molar bands

Simple randomization was conducted by one of the academic staff (not involved in the study) using computer-generated random numbers with an allocation ratio of 1 : 1. The allocation sequence was concealed using sequentially numbered, opaque, sealed envelopes opened only after obtaining the preoperative stereolithographic (STL) file.

### 2.4. Interventions

Comprehensive preoperative orthodontic records were obtained. The trial was conducted only on the maxillary dentition to eliminate any confounding factors that could be attributed to the difficulty in isolating the mandibular dentition. Alginate impressions (Zhermack® Orthoprint, Germany) were taken of the maxillary arches that were then poured with type IV extra hard stone to make the working models.

#### 2.4.1. Laboratory Stage

**Figure 1:**
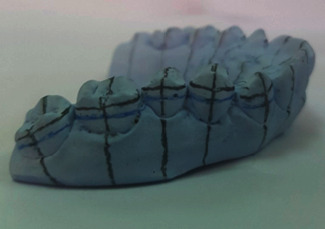
Horizontal and vertical reference lines.

**Figure 2:**
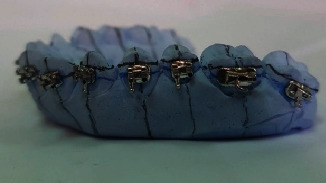
Unaltered orthodontic attachments group (UA).

**Figure 3:**
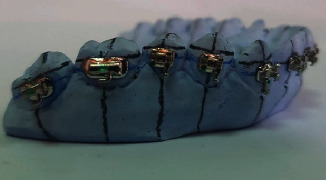
Custom composite resin base attachment group (CBA).

**Figure 4:**
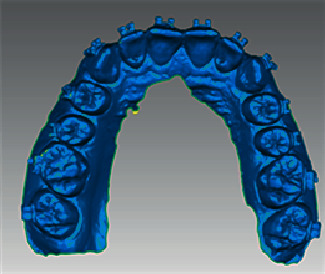
Preoperative model STL file.

Vertical and horizontal lines were then drawn on the working models. The vertical lines represented the long axes of the teeth, and in two horizontal lines: one represented the marginal ridges and the other one represented the buccal pit of the first molars. With the aid of a bow divider, the distance between the two lines was replicated to the rest of the teeth, noting that the space was decreased by 0.5 mm for the second molars (Figure 1). The orthodontic attachments (American Orthodontics, Master Series® TM 22″ Roth prescription) were attached to one side of the model with a thin layer of a water-soluble glue adhesive (Aleene's® All Purpose Tacky Glue, USA) to form the unaltered base attachment (UA) (Figure 2). The model's contralateral side was first coated with a separating medium (Sultan® denture separating medium, USA). The orthodontic attachments were subsequently attached to the model with a light-cured composite resin adhesive (Resilience® LC Orthodontic Adhesive) and cured with a dental curing light (Woodpecker® I led max, China) for 20 seconds per attachment to form the composite resin custom base attachment (CBA) (Figure 3). The working model was lightly sprayed with titanium dioxide powder (CEREC® Optispray, Sirona, Germany) to eliminate metallic reflections during digital scanning with an intraoral scanner (CEREC®, Omnicam AC, Sirona, Germany). The scanning mode was done by scanning the lingual, buccal, and occlusal sides, respectively, under room lighting (top level fluorescent lamps). The STL file was exported from the scanner software and regarded as the preoperative STL file (Figure 4).

**Figure 5:**
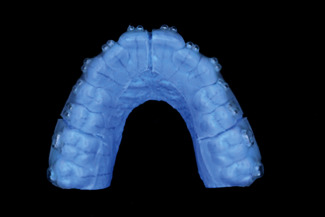
The vacuum-formed transfer tray after being cut into four halves.

A soft 1.5 mm thick sheet material was pulled over the working model using a vacuum forming machine (MiniStar S®, Scheu Dental Technology, Germany) and left to cool. The tray was then detached with the attachments in place, trimmed, and cleaned with a toothbrush. Interdental vertical cuts were made to facilitate tray removal from the patient's mouth (Figure 5). Next, all attachments were gently micro-etched with 50-micron aluminum powder (Danville Microetcher® II, CA, USA) under 75 psi air pressure to remove any plaster remnants and reactivate the CBAs.

#### 2.4.2. Clinical Stage

**Figure 6:**
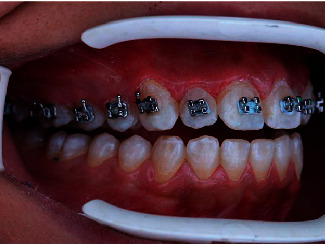
Postoperative view immediately after transfer tray removal.

Teeth were cleaned with pumice paste (Ultrapro® Tx Prophy Paste, USA), etched with 37% phosphoric acid gel (FineEtch®, UC dental products), and the orthodontic bonding agent (Transbond® XT (3M, Monrovia, CA, USA)) was placed on the enamel surface. For the UA group, a thin layer of light-cured adhesive resin was added directly to the attachments. In the CBA group, the bonding agent was added onto the composite resin custom bases and then directly inserted onto the patient's teeth. All attachments were cured for 20 seconds before tray detachment (Figure 6). The CBA technique does not allow for excess composite around the attachments; however, for the UA group, any excess composite was removed using a tungsten carbide bur (123-603-00, Dentaurum®, Pforzheim, Germany) in a slow speed handpiece after tray detachment and prior to scanning.

**Figure 7:**
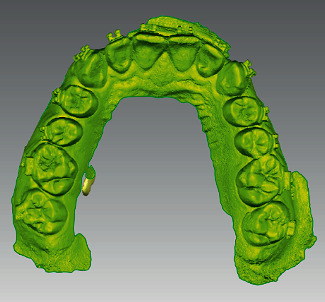
Postoperative model STL file.

In case an attachment failed during the bonding stage, the transfer tray was sectioned to include only the said attachment, and the attachment(s) bonded indirectly again in the same manner and were included in the statistical analysis. Transfer trays were then disinfected using a chlorhexidine gluconate antimicrobial agent and stored in a sterile box for each participant. Participants were then intraorally scanned with the same intraoral digital scanner to make the postoperative STL file (Figure 7).

**Figure 8:**
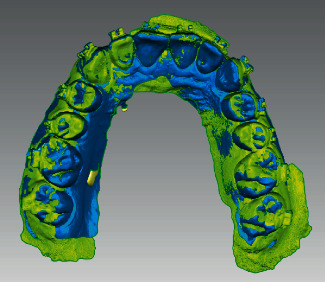
Superimposition of the preoperative and postoperative STL files.

**Figure 9:**
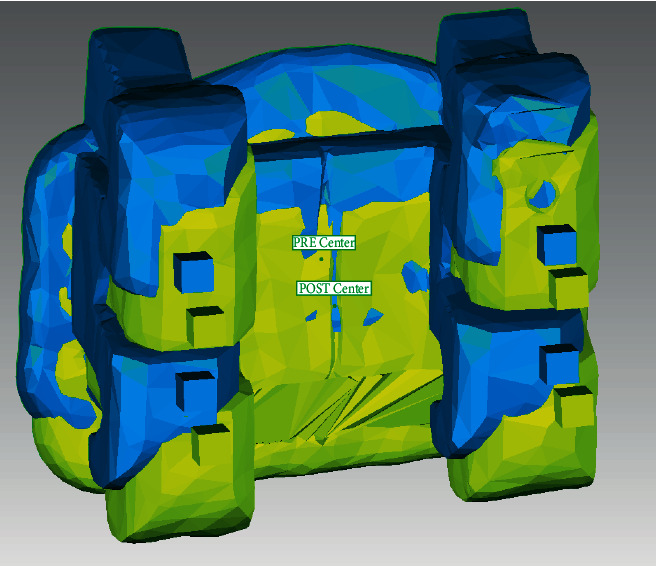
Superimposition of the precenter and postcenter coordinate systems.

The preoperative and postoperative scans were superimposed using Geomagic® Qualify software (Figure 8). The coordinate system of each attachment was set at its exact center, where the preoperative and postoperative coordinate systems were termed precenter and postcenter points, respectively (Figure 9).

### 2.5. Outcomes Measures

**Figure 10:**
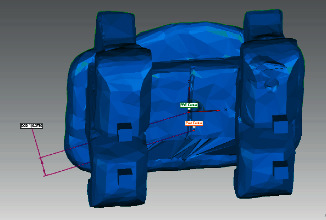
Measurement of the linear deviation between the preoperative and postoperative STL files.

**Figure 11:**
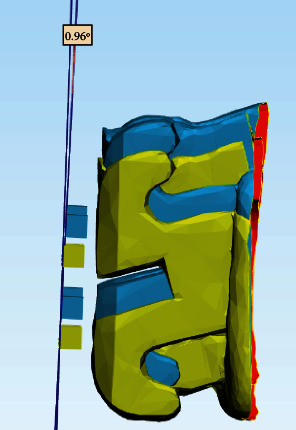
Measurement of the angular deviation (torque difference) between the superimposed models.

**Figure 12:**
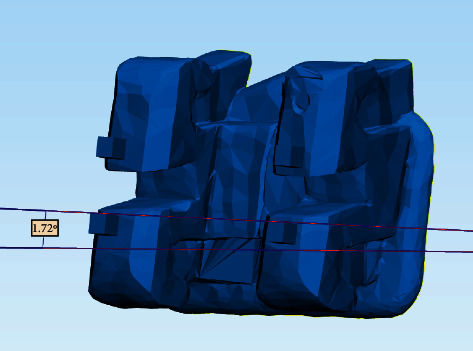
Tipping differences between the superimposed models.

**Figure 13:**
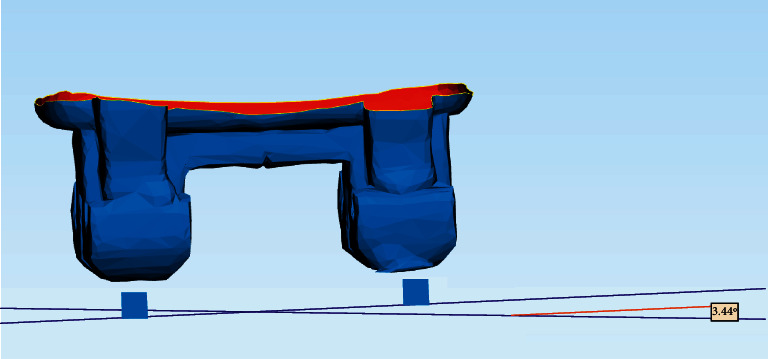
Rotational differences between the superimposed models.

The chairside time required to bond the attachments was measured for both groups and recorded in minutes. Attachment bond failure was recorded as the number of failed attachments immediately after attachment seating and tray removal. Finally, orthodontic attachment bonding accuracy was measured using three-dimensional scanning and superimposition (Figures 10–13).

### 2.6. Blinding

Both the orthodontist treatment provider and orthodontic patients were not possible to be blinded due to the nature of the study. However, all measurements and the statistical analysis were conducted blindly.

### 2.7. Statistical Analysis and Error of the Method

Statistical analysis was performed with Statistical Package for Social Science (SPSS) 20®, Graph Pad Prism®, and Microsoft Excel 2016. Data were presented as counts, percentages, means, and standard deviation (SD) values. Frequency statistics were conducted to describe the directional bias and frequency of error during indirect attachment placement; comparisons between the two groups for attachment bond failure and directional bias were performed using chi-square tests. Means and standard deviation values were explored for normality using the Shapiro–Wilk normality test, and then independent *t*-tests were performed for parametric data. The significance level was set at *P* < 0.05. Intrarater reliability was tested by repeating the measurements one month after the data collection. In order to determine the error of the method, the Intraclass Correlation Coefficient (ICC) was adopted.

## 3. Results

With regard to the intrarater reliability test, the ICC test showed a high level of agreement (0.9). The mean chairside times were 7.96 and 7.77 minutes for the UA and CBA groups, respectively. There was no significant difference between both groups (*P*=0.87) when employing independent *t*-tests. Concerning the attachment failure rate, for the UA group, there were 3.33% bracket failures and 16.66% tube failures. While for the CBA group, there were 10% bracket failures and 16.66% tube failures. Performing chi-square tests between both groups showed no statistically significant difference (*P*=0.37).

In terms of the accuracy of bonding, results are divided into linear and angular discrepancies, as follows:

As for linear discrepancies, starting with the total movement discrepancy, there was no statistically significant difference between both groups for all teeth (*P*=0.73). However, in the UA group, there was more accuracy in attachment placement for the maxillary first premolars and first molars (*P* < 0.01, *P*=0.03, respectively) and less accuracy for maxillary lateral incisors (*P*=0.01) than in the CBA group. Regarding mesiodistal discrepancy in the *X*-axis, there was no statistically significant difference between the two groups for all teeth (*P*=0.10). However, the UA technique was more accurate with maxillary first premolars (*P*=0.01) and less accurate for maxillary central incisors and first molars attachment placement (*P* < 0.01) than the CBA group. Considering vertical discrepancies in the *Z*-axis, there was no statistically significant difference between the two groups for all teeth (*P*=0.31). The UA method did show greater accuracy for the maxillary first premolar and first molar attachment placements (*P* < 0.01, *P*=0.04, respectively) and was less accurate for the upper central and lateral incisor (*P* < 0.01), and second premolar attachment placements (*P*=0.01) than the CBA group. Considering buccopalatal discrepancies in the *Y*-axis, the UA group was significantly more accurate than the CBA group (*P*=0.04), as listed in Table 1.

With regard to linear movement directional bias, chi-square tests showed no significant differences in the mesial or distal directions. Statistically significant differences were, however, found for most of the attachments as a directional bias towards the occlusal (*P* < 0.01) and palatal directions within the UA group only (*P*=0.02), as listed in Table 2.

While for angular discrepancies, beginning with the tip difference, the CBA group showed statistically significantly greater accuracy (*P* < 0.01) than the UA group, as shown in Table 3. There was no statistically significant difference between the two groups for all teeth for torque differences (*P*=0.21). However, the UA group showed greater accuracy for the maxillary central incisors (*P* < 0.01), while it was less accurate for maxillary first premolars (*P* < 0.01) and second molars (*P* < 0.01) than the CBA group. When considering rotational differences, there was no significant difference between the two groups (*P*=0.18). However, the UA group was more accurate, with a significant difference for upper canines (*P* < 0.01), first premolars (*P* < 0.01), and second molars (*P*=0.01). At the same time, it was less accurate for upper central incisors (*P*=0.02), second premolars (*P* < 0.01), and first molars (*P* < 0.01), with a significant difference to the CBA group.

In terms of the angular movement directional bias, chi-square tests showed no significant difference for tipping and torque bias between the two groups and within each group (*P* > 0.05). Whilst for rotational directional bias, the UA group showed more mesial-out deviation, with a statistically significant difference (*P*=0.02), as listed in Table 4.

## 4. Discussion

Indirect bonding has been introduced with the aim of providing a more precise attachment position, among other potential advantages. Orthodontic attachments can be bonded to the dental cast via a water-soluble glue (unaltered attachment) [3] or an adhesive-filled resin (custom composite base attachment) [15]. Many studies have tested the clinical effectiveness of different transfer methods [8–10]; however, the clinical and patient outcomes of the unaltered and custom composite base attachments have not been previously researched. Therefore, the present study aimed to investigate the clinical differences between the UA and CBA approaches during indirect attachment placement.

Regarding chairside time, the mean clinical time was not statistically significantly different between both groups, where it was approximately 8 minutes for bond-up per quadrant. Yildirim and Aydinatay [7] reported chairside time to be an average of 11–17 minutes per arch. As for attachment failure, there was no statistically significant difference between the two techniques, and the proportions were 3.33% and 10% immediately after removing the bracket seating trays for the UA and CBA techniques, respectively. These data are in agreement with the reported overall bond failure rate of Vijayakumar (8.80%) [16], Menini et al. (1.75%) [17], Grunheid et al. (9.80%) [10], and Yildirim and Aydinatay (10.71%) [7], whereas the overall bond failure was 11.25% in the study by Niu et al. [18]. Second molars were noted to have the highest detachment rate and are in agreement with Reed and O'Brien's study [19]. Moreover, during indirect bonding, it is practically difficult to insert a tray carrying multiple attachments to be bonded without having unequal pressure and inconsistent adhesive thickness, providing low bond strength [19]. Nevertheless, moisture contamination was thought to be generally low due to the tightly fitting transfer tray that provides an insulating zone for indirect bonding [10].

Regarding the placement of the coordinate system, they were chosen to be at the object's center of gravity, which is at the exact center of the orthodontic attachment. The reason for that is, from a biomechanical point of view, the center of gravity of an object would be the least point to be affected by the rotational movement if the object is rotating around this point. Any deviation at any axis would therefore be the result of a bodily movement on this axis and not due to rotation.

The present study used the method of determining accuracy as described by Elnigoumi [20] which was based on the reliability of three-dimensional (3D) models in terms of linear and angular measurements. For linear measurements, the present study showed no statistically significant differences between both indirect bonding methods for mesiodistal and vertical discrepancies; however, the UA technique was more accurate in terms of buccopalatal measurements. Castilla et al. [8] showed comparable results with the present study for linear deviations, while Schmid et al. [9] showed lower deviations for all linear measurements. Chaudhary et al. [12] found the majority of transfer errors were in the vertical dimension and that was more pronounced for 3D printed trays than for polyvinyl siloxane ones. As for linear directional bias, the UA technique showed more bias towards the occlusal (86%) and palatal (71%) directions due to the possible incomplete seating of the transfer tray and prolonged micro-etching of the orthodontic attachments, respectively. Occlusal bias was also noted in the various studies [8,12,18,21]. Other studies found a more directional bias towards the gingival [10] and buccal directions [10,12,18].

As for angular deviation, the CBA technique showed more accuracy (1.56 degrees) with statistical significance than the UA method (3.23 degrees). However, torque and rotational measurements showed comparable accuracy in the present study. Similar trends were found by Bachour et al. [13] where they found questionable angular dimension accuracy (more than 2-degree discrepancy). Nevertheless, it was noted in other studies with less tip, torque, and rotational deviations, and this could have been attributed to the low molar sample size of the former study (10 orthodontic tubes) and the in vitro design of the latter trial [9,10]. Regarding angular directional bias, there was more deviation towards the mesial-out direction for the UA technique. Similar trends were observed in the study by Niu et al. [18]. One hypothesis for this could be due to the possibility of uneven adhesive thickness or uneven finger pressure on the transfer tray during clinical bonding.

One of the merits of using the CBA technique is the lack of excess resin material around the orthodontic attachment, whereas the excess resin was required to be removed very carefully when utilizing the UA technique. The customized bases of the CBA attachments have the resin already cured in its final form versus a clean base meshwork for the UA attachments, which requires the clinical removal of any excess resin. This can be regarded as one of the reasons why orthodontic therapy could contribute to enamel demineralization, by leaving excess adhesive resin around the orthodontic attachment that may eventually lead to dental decay. Many clinical protocols have been developed to mitigate this shortcoming through introducing a wide range of preventive products such as fluoride varnishes [22] and more recently by incorporating hydroxyapatite products in toothpaste where they manifest promising results [23].

The statistical null hypothesis can be accepted, as it is evident that there is no statistically significant difference in chairside time, bond failure, and accuracy of bonding between the custom composite resin base and the unaltered base in the indirect bonding technique.

### 4.1. Limitations

More efficient isolation control systems during the clinical indirect bonding stage could be advocated in clinical settings to reduce attachment failure rates. Furthermore, the orthodontic attachments were manually placed on the models so that there was an unavoidable margin of error in the present study. Hence, using a 3D scan to position the orthodontic attachments digitally could provide less laboratory stage duration and possibly a high accuracy of attachment positioning. Even though the scanning powder is advantageous in preventing metallic reflections during scanning, inappropriate layering might have created different thicknesses at some points of the orthodontic attachments, and hence, a compromise in the overall scanning quality might occur [24]. As for the bonding accuracy, there was some directional bias towards the palatal direction; this could be attributed to the increased duration of micro-etching the orthodontic attachment base. Blinding was neither applied to the operator nor participants of the present trial. Although attachment failure rate overtime was not investigated in this study to avoid some possible confounding factors throughout treatments, this may also be a limitation and possible area for further investigation. Finally, future studies would benefit from the recent technological advancements in scanning and 3D printing, assisting the clinical aspects of the bonding procedures.

## 5. Conclusion

Chairside time and attachment failure rate differences were comparable between the two indirect bonding techniques, and no statistically significant difference existed between both techniques in terms of mesiodistal and vertical linear deviations for almost all attachments. Nevertheless, the unaltered attachment proved to be more accurate than the custom base technique regarding buccopalatal linear deviation for almost all attachments, whereas more occlusal and palatal directional bias was noted for the unaltered attachment group.

The custom base technique proved to be more accurate in tipping deviation compared to the unaltered attachment; however, the two techniques proved to be comparable in terms of torque and rotational deviations.

Both techniques appeared to be comparable for the percentage of angular directional deviation except for the more mesial-out rotational bias within the unaltered attachment technique. Ultimately, both methods were clinically reliable, with no major disadvantages that would prevent their usage.

## Figures and Tables

**Table 1:** Buccopalatal linear deviation (*Y*-axis) for the orthodontic attachments.

Tooth number	*N*	Group				Mean difference	*P* value
		Group I (UA) in microns		Group II (CBA) in microns			
		*M*	SD	*M*	SD		
1	6	106.00	34.58	382.50	124.78	−366.50	0.01^*∗*^
2	6	130.00	42.41	99.50	32.46	30.50	0.19
3	6	141.00	46.00	267.00	87.10	−126	0.01^*∗*^
4	6	289.50	94.44	94.00	30.66	195.50	0.01^*∗*^
5	6	84.00	27.40	330.00	107.65	−246	0.01^*∗*^
6	6	49.50	16.15	111.80	36.47	−62.30	0.01^*∗*^
7	6	284.50	92.81	418.00	136.36	−133.50	0.07
Overall		154.93	50.54	243.26	79.35	−88.30	0.04^*∗*^

*N*: attachment count; *P* value: probability value; *M*: mean value; SD: standard deviation. ^*∗*^Significant difference.

**Table 2:** Linear directional bias of orthodontic attachments (*Y* and *Z* axes).

	Occlusal	Gingival	*P* value	Buccal-out	Palatal-in	*P* value
Group I (UA)	85.70%	14.20%	0.01^*∗*^	28.50%	71.40%	0.02^*∗*^
Group II ( CBA)	64.20%	35.70%	0.13	42.80%	57.14%	0.43
*P*-value	0.214	0.20		0.44	0.46	

*P* value: probability value. ^*∗*^Significant difference.

**Table 3:** Tipping (angular) deviation of orthodontic attachments.

Tooth number	*N*	Group				Mean difference	*P* value
		Group I (UA) in degrees		Group II (CBA) in degrees			
		*M*	SD	*M*	SD		
1	6	6.50	2.12	1.60	0.52	4.90	0.01^*∗*^
2	6	2.10	0.69	1.10	0.36	1	0.01^*∗*^
3	6	2.40	0.78	0.40	0.13	2	0.01^*∗*^
4	6	2.00	0.65	3.40	1.11	−1.40	0.02^*∗*^
5	6	4.50	1.47	1.90	0.62	2.60	0.01^*∗*^
6	6	0.50	0.16	0.60	0.20	−0.10	0.33
7	6	4.60	1.50	1.90	0.62	2.70	0.01^*∗*^
Overall		3.23	1.05	1.56	0.51	1.70	0.05^*∗*^

*N*: attachment count; *P* value: probability value; *M*: mean value; SD: standard deviation. ^*∗*^Significant difference.

**Table 4:** Rotational (angular) directional bias.

	Mesial out (+)	Mesial in (−)	*P* value
Group I (UA)	71.40%	28.60%	0.02^*∗*^
Group II (CBA)	42.80%	57.10%	0.43
*P* value	0.12	0.13	

*P* value: probability value. ^*∗*^Significant difference.

## Data Availability

The data supporting the study can be obtained directly from the corresponding author upon request.
